# Surgery After Thermal Ablation of Thyroid Nodules: A Systematic Review of Surgical Risk, Strategy and Pathological Findings

**DOI:** 10.1002/hed.70487

**Published:** 2026-09-27

**Authors:** Francesco Brucchi, Federica Poli, Beatrice Sperotto, Carla Colombo, Laura Fugazzola, Gianlorenzo Dionigi

**Affiliations:** ^1^ Department of Pathophysiology and Transplantation University of Milan Milan Italy; ^2^ Division of General Surgery Istituto di Ricovero e Cura a Carattere Scientifico (IRCCS) Istituto Auxologico Italiano Milan Italy; ^3^ Department of Endocrine and Metabolic Diseases, Endocrine Oncology Unit IRCCS Istituto Auxologico Italiano Milan Italy; ^4^ Endocrinology Unit ASST Santi Paolo e Carlo Milan Italy

**Keywords:** fibrosis, histopathology, laser ablation, radiofrequency ablation, surgical complications, systematic review, thermal ablation, thyroidectomy

## Abstract

**Background:**

Thermal ablation is increasingly used for selected thyroid nodules, but some patients subsequently require thyroidectomy. The effect of prior ablation on surgical risk, operative strategy, and histopathology remains unclear.

**Methods:**

PubMed/MEDLINE, EMBASE, and the Cochrane Library were searched from inception to May 2026 for studies reporting surgical and/or histopathological outcomes of thyroidectomy after radiofrequency, laser, or microwave ablation. Cohort studies and case series with at least two patients were included. Data were synthesized descriptively and stratified by ablation indication: benign/indeterminate nodules versus primary papillary thyroid carcinoma.

**Results:**

Nine retrospective studies including 150 patients were included: six on benign/indeterminate nodules (*n* = 105) and three on primary papillary thyroid carcinoma (*n* = 45). In benign/indeterminate cases, transient recurrent laryngeal nerve palsy ranged from 0% to 9.7%, transient hypoparathyroidism from 0% to 6.5%, and hematoma requiring reoperation from 0% to 6.5%; no permanent recurrent laryngeal nerve palsy or hypoparathyroidism was reported. Controlled studies showed longer operative times, higher thyroidectomy difficulty scores, and more severe adhesions after ablation, although overall complication rates did not differ significantly. Histopathology showed fibrosis, hyalinization, coagulative necrosis, and treatment‐related atypia, potentially complicating assessment of follicular‐patterned neoplasms; no carcinoma arising within the ablated zone was identified. In the papillary thyroid carcinoma group, surgical specimens frequently revealed residual tumor and lymph node metastases after ablation used as primary treatment, while oncological follow‐up was largely absent. Reporting of surgical strategy and intraoperative adjuncts was inconsistent.

**Conclusions:**

Thyroidectomy after thermal ablation appears feasible in experienced endocrine surgery centers, with low reported permanent morbidity, but may be technically more complex than primary surgery. Current evidence is limited to small, heterogeneous retrospective series and cannot define the true incremental surgical risk, optimal operative strategy, or long‐term oncological impact of prior ablation. Prospective multicenter comparative studies with standardized reporting are needed.

## 1 Introduction

Over the past decade, image‐guided thermal ablation has emerged as a minimally invasive alternative to surgery for selected thyroid lesions. International recommendations, including those of the European Thyroid Association and other multidisciplinary expert panels, now recognize radiofrequency ablation (RFA), laser ablation (LA), and microwave ablation (MWA) as effective and generally safe treatment options for appropriately selected patients with benign thyroid nodules and, in highly selected cases, for indeterminate lesions or limited low‐risk thyroid malignancy when surgery is undesirable, deferred, or contraindicated [1, 2, 3, 4, 5, 6]. This progressive expansion of indications has been driven by the attractive peri‐procedural profile of ablation, particularly its low reported rate of major complications, its feasibility as an outpatient or short‐stay procedure, and its ability to preserve thyroid parenchyma and avoid conventional surgery‐related morbidity.

Despite these advantages, clinical practice has increasingly identified a subset of patients for whom thermal ablation does not represent the end of treatment but rather an intermediate step before surgery. In the benign and indeterminate setting, delayed thyroidectomy may become necessary due to incomplete volume reduction, persistent or recurrent compressive or cosmetic symptoms, regrowth after an initial response, discordant imaging‐cytology findings, repeated nondiagnostic sampling, or cytological and/or molecular reclassification toward indeterminate, suspicious, or frankly malignant categories [7, 8, 9, 10]. In the oncological setting, where ablation has been explored more selectively and remains substantially more controversial, surgery may be required due to incomplete tumor eradication, persistence of viable neoplastic tissue, multifocal disease not adequately addressed by local treatment, unfavorable location, or subsequent recognition of cervical nodal metastases or other features that restore the indication for formal oncological resection.

These scenarios should not be conflated. Patients undergoing thyroidectomy after ablation for benign nodular disease and those referred to surgery after ablation of malignant or potentially malignant lesions differ fundamentally in baseline risk profile, therapeutic intent, extent of surgery required, pathological expectations, and oncological consequences of residual disease. They also differ in the threshold for reintervention, the acceptable degree of residual viable tissue, and the relevance of nodal assessment or compartment‐oriented surgery. For these reasons, benign or indeterminate post‐ablation surgery and oncological post‐ablation surgery should be interpreted as biologically and surgically distinct entities rather than as variations of the same clinical problem.

A central and still insufficiently addressed issue is that patients are often initially offered ablation on the basis of a very favorable safety profile, yet a proportion may later require thyroidectomy under conditions that are potentially more demanding than those of primary surgery. Thermal injury can induce coagulative necrosis, chronic inflammation, reparative fibrosis, scarring, and perithyroidal adhesions, thereby obscuring tissue planes and modifying the operative field [11, 12]. Emerging reports specifically describing thyroidectomy after prior ablation suggest that these tissue changes may translate into greater technical difficulty, more challenging recurrent laryngeal nerve (RLN) dissection, and less straightforward capsular mobilization, with possible implications for postoperative morbidity.

This issue is clinically relevant because thyroidectomy is generally a safe procedure in experienced hands, but it remains associated with well‐recognized complications, including RLN injury, postoperative hypoparathyroidism, cervical hematoma, and, in selected cases, the need for more extensive resection than originally anticipated [13, 14, 15]. Such complications may substantially affect swallowing, phonation, calcium homeostasis, long‐term quality of life, and patient‐reported satisfaction with care [13, 14, 15, 16, 17]. Accordingly, any prior intervention that has the potential to increase operative complexity, alter pathological interpretation, or affect perioperative outcomes deserves systematic evaluation.

From a clinical decision‐making perspective, this information is relevant not only to endocrine surgeons but also to endocrinologists, interventional radiologists, multidisciplinary tumor boards, and, importantly, to patients. Data on the operative complexity, morbidity profile, and histopathological findings of thyroidectomy following prior ablation are essential for counseling patients being considered for salvage surgery after RFA, LA, or MWA. Equally importantly, these data should inform the initial consent process before ablation, as a minimally invasive first‐line treatment cannot be discussed appropriately without acknowledging that delayed surgery, if required, may be technically different from primary thyroidectomy.

With the broader adoption of thyroid ablation, surgeons have increasingly encountered such post‐ablation surgical cases in routine practice, and the literature has begun to reflect this development through dedicated reports focusing on operative difficulty, fibrosis‐related risk, and pathological correlates. However, the available evidence remains limited and methodologically heterogeneous, consisting mainly of retrospective, single‐center experiences with small sample sizes, mixed indications, different ablation technologies, and variable follow‐up intervals.

To date, no systematic review has specifically synthesized the surgical and pathological outcomes of thyroidectomy performed after prior thyroid thermal ablation. The objective of this study was to systematically examine the emerging evidence on thyroidectomy following prior thermal ablation, with particular emphasis on indications for delayed surgery, surgical strategy, operative difficulty and morbidity, and the pathological substrate underlying post‐ablation surgical risk.

In view of the emerging but still fragmented literature on surgery after thyroid nodule ablation, this review was designed not only to summarize available data, but also to map the global evidence landscape, delineate recurring patterns, and identify areas where information is critically lacking. This dual descriptive and exploratory objective is consistent with contemporary scoping review methodology and with the need to inform future prospective, multicenter research in an evolving field.

## 2 Materials and Methods

### 2.1 Protocol and Registration

This systematic review is reported in accordance with the Preferred Reporting Items for Systematic Reviews and Meta‐Analyses (PRISMA) 2020 statement [18].

The review protocol was prospectively registered in the PROSPERO database (registration ID: CRD420261405826).

### 2.2 Eligibility Criteria (PICO Framework)

#### 2.2.1 Population

Adult patients (≥ 18 years) with thyroid nodules (benign, indeterminate, or low‐risk malignant) who had undergone at least one session of thermal ablation and subsequently received thyroid surgery.

#### 2.2.2 Intervention

Prior percutaneous thermal ablation of one or more thyroid nodules (RFA, LA, or MWA), with no restriction on the number of sessions.

#### 2.2.3 Comparator

Where available, a control cohort of patients undergoing thyroidectomy without prior ablation. Studies without a comparator group were also eligible provided they reported quantitative surgical and/or histopathological outcomes.

#### 2.2.4 Outcomes

Primary outcomes focused on surgical risk and included RLN palsy (transient and permanent), postoperative hypoparathyroidism, and cervical hematoma requiring re‐intervention. In addition, we evaluated indicators of surgical complexity, such as operative time, the presence and severity of adhesions or fibrosis, incidental parathyroidectomy, and any formal or surrogate measures of surgical difficulty. To capture the surgical strategy adopted in postablation settings, we collected data on the extent of resection (total thyroidectomy versus hemithyroidectomy), the use of staged procedures, the application of intraoperative neuromonitoring (IONM) and near‐infrared fluorescence (NIRF) for parathyroid identification, the interval between ablation and surgery, and whether centers explicitly declared high‐volume endocrine surgery activity. Finally, we systematically recorded pathological and oncological outcomes, including detailed histopathological changes (fibrosis, necrosis, residual tumor, and cytological or architectural alterations), final pathological diagnosis, presurgical nodule volume reduction rate (VRR), preoperative ultrasound characteristics, and, where available, postoperative oncological follow‐up in patients operated on for malignant disease.

#### 2.2.5 Study Design

Original research articles (randomized controlled trials, prospective or retrospective cohort studies, and case series with *n* ≥ 2 patients) published in peer‐reviewed journals in English.

#### 2.2.6 Exclusion Criteria

Review articles, editorials, letters to the editor, single case reports (*n* = 1), conference abstracts without full text, studies dealing exclusively with thermal ablation of parathyroid tissue or nonthyroid cervical lesions, studies focusing solely on ablation of locoregional recurrences or cervical lymph node metastases without data on primary thyroid nodules, and studies not reporting any quantitative surgical or histopathological outcomes.

### 2.3 Information Sources and Search Strategy

A systematic literature search was performed in PubMed/MEDLINE, EMBASE, and the Cochrane Library from database inception to May 25, 2026. The search strategy combined Medical Subject Headings (MeSH) and free‐text terms related to thermal ablation (RFA, LA, MWA), thyroid nodules, thyroidectomy, surgery, complications, and pathology.

Reference lists of all included studies and relevant review articles were also manually screened to identify additional eligible studies. The complete search strategies, including exact search strings and Boolean operators for each database, are reported in Appendix S1 .

### 2.4 Study Selection and Data Extraction

Titles and abstracts were screened independently by two reviewers (F.P. and F.B.) using a standardized screening form. Full texts of potentially eligible articles were then assessed in detail. Disagreements at either stage were resolved by discussion and, if necessary, by consultation with a third reviewer (G.D.).

Data were extracted by one reviewer and independently verified by a second reviewer using a prespecified extraction form. Extracted variables included: first author, year of publication, country, study design, sample size, ablation modality, ablation indication, surgical indication, time interval between ablation and surgery, type and extent of thyroidectomy, intraoperative findings (including adhesions/fibrosis when reported), postoperative complications (with explicit specification of denominators: per patient, per lobe, or per nerve at risk), operative time, and histopathological findings. Additionally extracted, where available, were use of IONM; use of NIRF imaging; type of thyroidectomy (total versus hemithyroidectomy); occurrence of staged thyroidectomy; presurgical nodule VRR; presurgical ultrasound pattern; definition and measurement of surgical difficulty (validated scale, surrogate measure, or qualitative assessment); explicit declaration of center volume; and postoperative oncological follow‐up duration and outcomes in patients operated for malignant disease.

### 2.5 Risk of Bias Assessment

Risk of bias was assessed independently by two reviewers using the Newcastle–Ottawa Scale (NOS) for cohort studies [19]. The NOS evaluates three domains (Selection, Comparability, and Outcome) with a maximum total score of 9 points. Studies were classified as having low risk of bias (≥ 7 points), moderate risk of bias (5–6 points), or high risk of bias (≤ 4 points).

For single‐arm studies without a nonablated comparator group, comparator‐related NOS items were, as prespecified, considered not fulfilled. Discrepancies between reviewers were resolved by consensus. Item‐level scores, total NOS scores, corresponding risk‐of‐bias categories, and justifications for each judgment are reported in Appendix S2.

### 2.6 Data Synthesis

Given the substantial clinical and methodological heterogeneity across the included studies—including differences in ablation modalities, patient populations (benign versus malignant nodules), indications for surgery, outcome definitions, follow‐up duration, and denominator conventions—a formal meta‐analysis was deemed inappropriate. Results are therefore presented as a descriptive, narrative synthesis structured around the two main clinical scenarios (benign/indeterminate nodules versus primary papillary thyroid carcinoma [PTC]).

Outcome data are reported as proportions with explicit numerators and denominators. Where two or more studies provided comparable data with matched control groups, the results of direct comparisons between ablated and nonablated cohorts are summarized narratively. The frequent occurrence of multiple zero‐event studies for key outcomes (RLN palsy, cervical hematoma, and hypoparathyroidism) further precluded robust quantitative pooling and is acknowledged as a relevant limitation of the available evidence.

Given the marked clinical and methodological heterogeneity and the small, retrospective nature of the available series, this review was deliberately conceived as a descriptive, mapping‐oriented synthesis. The aim is to systematically characterize the existing evidence and its gaps rather than to produce definitive pooled estimates of surgical risk or oncological outcomes.

## 3 Results

### 3.1 Study Selection

The systematic search of electronic databases and registers retrieved a total of 5246 records. After removal of 1615 duplicate records, 3631 records were screened by title and abstract. Of these, 3610 records were excluded, and 21 reports were sought for retrieval. All 21 reports were successfully retrieved and assessed for eligibility. Twelve reports were excluded after full‐text assessment: eight because of wrong population, three because of wrong study design, and one because it was available only as an abstract/poster. Overall, nine studies met the inclusion criteria and were included in the review. The PRISMA flow diagram is presented in Figure 1.

**FIGURE 1:**
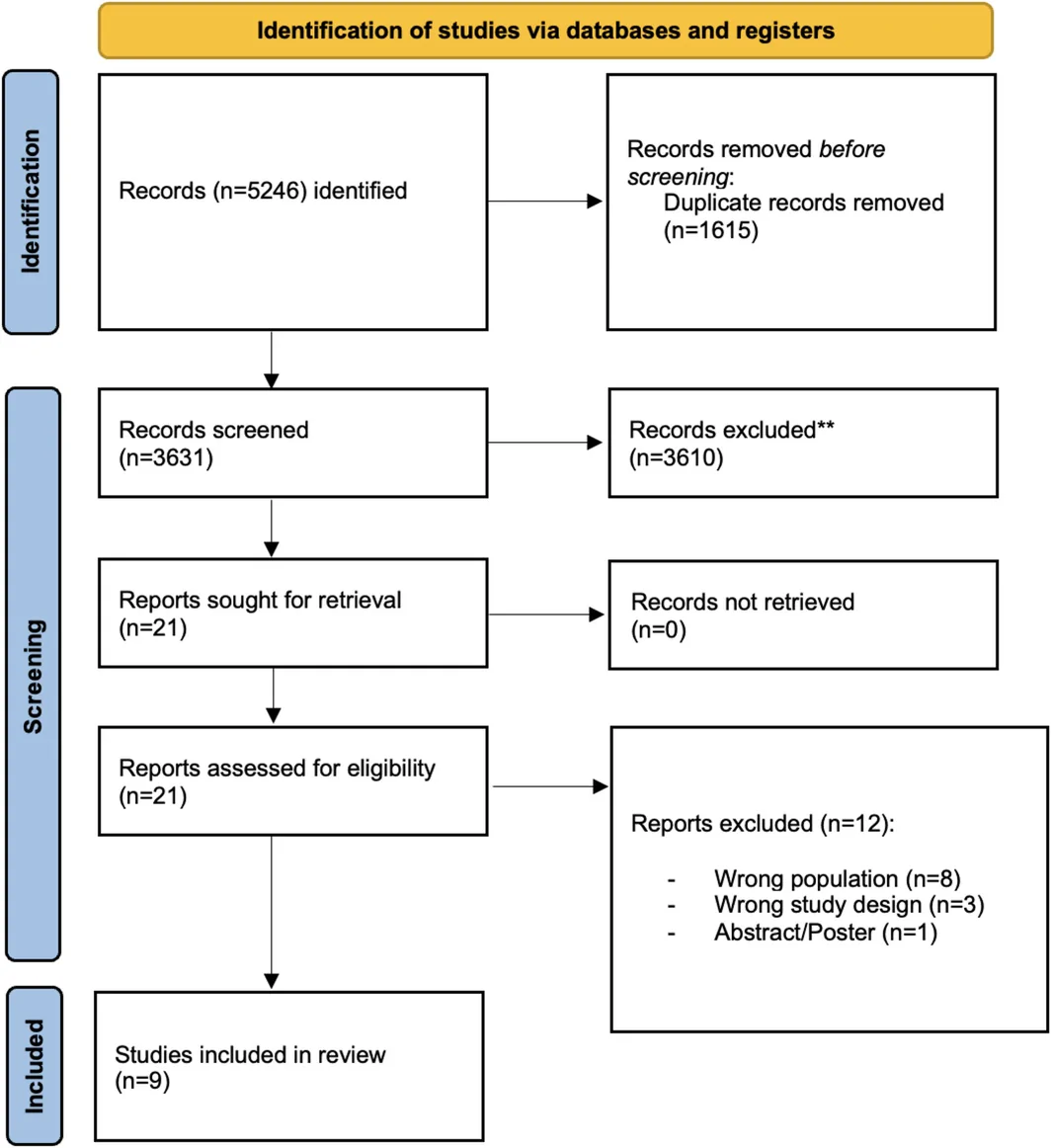
PRISMA 2020 flow diagram.

### 3.2 Study Characteristics and Risk of Bias

All nine included studies were retrospective in design, published between 2012 and 2026 and representing a total of 150 patients who underwent thyroidectomy after prior thermal ablation. Studies were conducted in Italy (*n* = 3), China (*n* = 3), Taiwan (*n* = 1), the United States (*n* = 1), and a joint Italian‐Chinese collaboration (*n* = 1). Individual study sizes ranged from 2 to 31 patients, with a median of 20 patients per study. For analytical and interpretive purposes, included studies were classified into two groups based on the primary indication for ablation:

*Group A*—Benign or indeterminate thyroid nodules: six studies, *n* = 105 patients [11, 12, 20, 21, 22, 23].

*Group B*—Primary PTC: three studies, *n* = 45 patients [24, 25, 26].

Ablation modalities represented included RFA (six studies), LA (two studies), MWA (one study), and combined modalities (two studies). No randomized controlled trials were identified. Study characteristics are summarized in Table 1.

**TABLE 1:** Characteristics of included studies, stratified by clinical scenario.

First author	Year	Design	Country	*N*	Ablation	Indication for ablation	Indication for surgery	Ablation‐to‐surgery interval
*Group A—Benign/indeterminate nodules*								
Piana et al.	2012	Retro case series	Italy	22	LA	Benign	Treatment failure (*n* = 17); indeterminate cytology Thy3 (*n* = 5)	Mean 38.6 months (range 2–74)
Russotto et al.	2024	Retro inst. exp.	Italy	7	LA	Benign	Nodule regrowth after TA	NR
Dobrinja et al.	2015	Retro cohort	Italy	2	RFA	Thy3 (follicular lesion)	Nodule regrowth (malignancy suspected)	Mean 15 months (range 6–24)
Zhang et al.	2026	Retro cohort	Italy/China	31	RFA/EtOH	Benign/low‐risk	Regrowth/symptoms/suspicious cytology/graves’	Mean 43.9 months (median 35)
Hussein et al.	2024	Retro comparative	USA	23	RFA	Benign	Suboptimal VRR/regrowth/positive mol. testing/toxic nodule	Median 6.7 months (3.5–13.4)
Kuo et al.	2024	Retro cohort	Taiwan	20	RFA	Benign	Regrowth/suboptimal VRR/suspicious US/follicular neoplasm	Mean 29.5 months (range 3.9–129)
*Group B—Primary papillary thyroid carcinoma (PTC)*								
Ding et al.	2022	Retro multicenter	China	12	MWA	Primary PTC	New PTC/suspected recurrence/LNM/patient preference	Median 6.6 months (0.4–21.9)
Ma et al.	2018	Retro case review	China	12	RFA/MWA/LA	Primary PTC	Suspicious US/confirmed malignancy/LNM/patient preference	NR
Sun et al.	2020	Retro single‐ctr	China	21	RFA	Primary PTC	Suspicious US or CT findings/LNM/patient preference	Median 30 days (range 6–368 days)

Abbreviations: EtOH = percutaneous ethanol injection; LA = laser ablation; LNM = lymph node metastasis; MWA = microwave ablation; *N* = number of patients undergoing postablation thyroidectomy; NR = not reported; PTC = papillary thyroid carcinoma; RFA = radiofrequency ablation; UMP = uncertain malignant potential; VRR = volume reduction rate.

A clear temporal trend in publication activity was evident: two of the nine included studies were published between 2012 and 2021, whereas five were published between 2022 and 2026, including three in 2024 alone. This pattern is consistent with the progressive consolidation of thermal ablation as a standard clinical practice and the consequent emergence of sufficiently large post‐ablation surgical series to justify dedicated publications.

Most studies carried at least moderate overall risk of bias, predominantly driven by their retrospective design, small sample sizes, and the lack of a matched control group in most series (Appendix S2). Only two studies [11, 12] included a contemporaneous nonablated comparator group and used prospectively collected registry data, resulting in a relatively lower overall risk of bias. The remaining studies were single‐arm series, with selection of patients for postablation surgery representing the principal source of selection bias (patients requiring surgery after ablation are not representative of all ablated patients). Outcome ascertainment was generally adequate in studies reporting IONM and systematic histopathological evaluation; it was judged as high risk in studies relying solely on clinical assessment without standardized protocols.

With regard to center volume, only one study [23] explicitly declared operating in a high‐volume center; Kuo et al. [12] implicitly indicates high surgical volume through its large contemporaneous control cohort (*n* = 145). The remaining seven studies did not formally declare center volume.

### 3.3 Group A—Thyroidectomy After Ablation for Benign or Indeterminate Nodules

#### 3.3.1 Surgical Outcomes

Across the six Group A studies (total *n* = 105), postoperative complication rates were generally low, although interstudy comparison is limited by differences in outcome definitions, denominators, and follow‐up duration.

##### 3.3.1.1 Operative Time and Surgical Difficulty

Two studies provided direct comparisons with nonablated control groups. Kuo et al. [12] (*n* = 20 vs. *n* = 145 controls) reported a significantly longer operative time in the ablation group (mean 99.5 vs. 69.5 min; *p* = 0.010), a higher thyroidectomy difficulty scale (TDS; median 9 vs. 6; *p* < 0.001), and more severe macroscopic adhesion scores (MAS grade ≥ 3: anterior 50% vs. 16.6%, *p* < 0.001; posterior 35.0% vs. 16.6%, *p* = 0.048). Hussein et al. [23] (*n* = 23 vs. *n* = 73 controls) observed a numerically longer operative time in post‐RFA patients (median 110 vs. 92 min), although this difference did not reach statistical significance (*p* = 0.40), possibly reflecting a Type‐II error given the limited sample size and the high‐volume center experience of the operating surgeons. Zhang et al. [11] described perithyroidal adhesions and fibrotic remodeling as consistent intraoperative findings, while Dobrinja et al. [22]—reporting only two cases—found no macroscopic perithyroidal fibrosis at surgery, though these results should be interpreted with extreme caution given the very limited sample.

##### 3.3.1.2 Recurrent Laryngeal Nerve Palsy

RLN palsy was reported in all six studies. Among the four studies with zero events [12, 20, 21, 22], the absence of RLN injury likely reflects both the generally benign nature of the procedures and the small sample sizes, which preclude reliable estimation of true rates. Zhang et al. [11] reported transient RLN palsy in 3/31 patients (9.7%), with intraoperative loss of signal (LOS) in 5/47 nerves at risk (10.6%). Hussein et al. [23] documented transient RLN injury or hoarseness in 2/23 post‐RFA patients (8.7%), a rate statistically comparable to the 8.2% observed in non‐RFA controls (*p* = 0.94). No permanent RLN palsy was reported in any Group A study.

##### 3.3.1.3 Postoperative Hypoparathyroidism and Incidental Parathyroidectomy

Hypoparathyroidism was reported in three Group A studies: Zhang et al. [11] documented transient hypoparathyroidism in 2/31 patients (6.5%, all recovered within 6 months), Hussein et al. [23] reported no cases in the post‐RFA group, and Kuo et al. [12] reported no cases of clinical hypocalcemia, though they documented a significantly higher rate of incidental parathyroidectomy in the ablation group (3/28 lobes, 10.7%) compared with controls (3/185 lobes, 1.6%; *p* = 0.031). This finding suggests that perithyroidal adhesion may compromise parathyroid preservation independently of clinical hypoparathyroidism rates. Piana et al. [20], Russotto et al. [21], and Dobrinja et al. [22] reported no hypoparathyroidism, although these studies were based on small samples with limited statistical informativeness.

##### 3.3.1.4 Postoperative Hematoma

Hematoma requiring reoperation was reported in two studies: Zhang et al. [11] (2/31 patients, 6.5%) and Hussein et al. [23] (1/23 patients, 4.3%, not significantly different from controls, *p* = 0.07). No hematoma was reported in the remaining four studies, though the small sample sizes preclude meaningful interpretation of these zero‐event results.

#### 3.3.2 Surgical Strategy

##### 3.3.2.1 Extent of Resection

The extent of thyroidectomy was not systematically specified in most Group A studies. Kuo et al. [12] was the only study to report this explicitly, indicating that 12 of 20 patients (60%) underwent hemithyroidectomy and 8 (40%) total thyroidectomy, as inferred from 28 lobes at risk in 20 patients. In the remaining five Group A studies, total thyroidectomy is either implied or not clearly reported. No study in either Group A or Group B described the use of staged thyroidectomy.

##### 3.3.2.2 Nerve Monitoring

Use of IONM was explicitly documented in three of the six Group A studies. Zhang et al. [11] reported intraoperative LOS in 5 of 47 nerves at risk (10.6%), and Hussein et al. [23] observed LOS in 3 of 23 patients (13.0%); in Kuo et al. [12], routine IONM use is implied by the denominator of 28 nerves at risk in 20 patients. Piana et al. [20], Russotto et al. [21], and Dobrinja et al. [22] did not mention IONM, and none of the three Group B studies reported its use.

##### 3.3.2.3 Fluorescence

NIRF imaging for intraoperative identification or perfusion assessment of the parathyroid glands was not reported in any of the nine studies.

##### 3.3.2.4 Nodule Volume Reduction

The pre‐surgical nodule VRR was not provided as an aggregate quantitative variable in any included study. In Hussein et al. [23] and Kuo et al. [12], suboptimal VRR was listed among the indications for surgery, but mean or median values were not reported. No study offered a formal, standardized preoperative ultrasound characterization of the nodule (e.g., TIRADS classification). Suspicious ultrasound features were cited as an indication for surgery in five studies, but ultrasound patterns were not coded in a reproducible way.

##### 3.3.2.5 Surgical Difficulty

The definition and measurement of surgical difficulty were heterogeneous. Only Kuo et al. [12] used validated instruments, namely the TDS (median 9 vs. 6, *p* < 0.001) and the Macroscopic Adhesion Score. In Hussein et al. [23] and Zhang et al. [11], operative time served as a surrogate marker of difficulty. In Dobrinja et al. [22] and in all three Group B studies, surgical difficulty was assessed solely through qualitative descriptions of perithyroidal adhesions, whereas Piana et al. [20] and Russotto et al. [21] did not address this outcome.

##### 3.3.2.6 Surgical Access

A further common limitation is that none of the studies specified the cervical approach (standard midline access versus a lateral “back‐door” approach to the prethyroidal strap muscles). It is therefore unclear whether surgeons deliberately modified the route of access to deal with postablative fibrosis. In addition, no study reported the use of minimally invasive, endoscopic, or robotic techniques: all procedures appear to have been performed through conventional open surgery. The lack of detail on the surgical approach precludes any assessment of how different access strategies might influence operative difficulty and risk in the postablation setting.

#### 3.3.3 Histopathological Findings—Benign/Indeterminate Group

Histopathological data were reported in five of the six Group A studies (Russotto et al. [21] did not present detailed histopathological characterization beyond final diagnosis).

##### 3.3.3.1 Fibrosis and Tissue Remodeling

Fibrosis or hyalinization was the most consistently reported finding across all studies. Piana et al. [20] described a temporal evolution of ablation‐induced changes: within 2 months of LA, the ablated area contained dark amorphous material surrounded by acute inflammatory infiltrate (neutrophils, lymphocytes, macrophages); after 18 months or more, the inflammation had resolved and been replaced by a well‐circumscribed area of dense fibrous tissue. Zhang et al. [11] quantitatively assessed the three main tissue components within ablated nodules: mean sclerosis 19.2%, mean coagulative necrosis 14.6%, and mean residual viable thyroid parenchyma 66.1%. These proportions exhibited substantial interpatient variability. Kuo et al. [12] found acellular hyalinization in 19/20 cases (95%), chronic inflammation in 16/20 (80%), and coagulative necrosis in 11/20 (55%). Dobrinja et al. [22] described central, scattered areas of hyaline sclerosis that did not involve the nodule capsule and did not impair diagnostic assessment in their two cases.

##### 3.3.3.2 Cytological Atypia and Diagnostic Challenges

Ablation‐induced cellular alterations that may complicate histopathological interpretation were documented in several studies. Kuo et al. [12] identified treatment‐related cytological changes—including nuclear enlargement, focal chromatin clearing, clear‐cell change, and atypical cyst lining—in 6/20 cases (30%), predominantly in follicular cells within or adjacent to the ablated zone. These changes may superficially resemble features associated with PTC, including nuclear grooves and chromatin clearing, and may therefore mislead cytopathological or histopathological interpretation if ablation history is not communicated. Piana et al. [20] similarly noted that 5 of 16 patients undergoing post‐LA fine‐needle aspiration (FNA) received an indeterminate (Thy3) cytological report, all of which corresponded to benign lesions on histopathology. Russotto et al. [21] reported focal HBME‐1 immunoreactivity in one of seven nodules, limited to an area of structural disarray and nuclear atypia, with all other malignancy markers (galectin‐3, BRAF V600E, p53) remaining negative and a Ki‐67 labeling index below 3% in all cases, consistent with ablation‐related—rather than neoplastic—changes.

##### 3.3.3.3 Follicular Neoplasm Assessment Challenges

Kuo et al. [12] documented, particularly, significant diagnostic challenges in eight patients with follicular neoplasms on final histopathology. In five of eight cases, assessing capsular integrity was complicated by extensive stromal hyalinization and tissue fragmentation, which either obscured or simulated capsular invasion. In one case, tumor identity and clonality were established by positive NRAS Q61R immunostaining. Obliterated peritumoral vessels in 5/8 cases raised further uncertainty regarding true vascular invasion. Three cases were ultimately classified as follicular carcinoma, and two as follicular tumors of uncertain malignant potential. These findings underscore the importance of informing the pathologist of the patient's ablation history and of judiciously employing ancillary immunohistochemical and molecular markers in equivocal cases.

##### 3.3.3.4 Final Diagnosis and Absence of Ablation‐Induced Malignant Transformation

No study identified thyroid carcinoma arising within the ablated zone, and there was no histopathological evidence of malignant transformation directly attributable to thermal ablation. Incidental papillary microcarcinomas were identified in two patients in the series by Piana et al. [20] and in three patients (two micro‐PTC and one medullary carcinoma) by Zhang et al. [11], all located at sites topographically distinct from the ablated nodule and consistent with background prevalence of occult thyroid cancer. However, the available evidence on this specific question is insufficient for definitive conclusions.

### 3.4 Group B—Surgery After Ablation for Primary PTC

#### 3.4.1 Surgical Outcomes—PTC Group

Three studies specifically evaluated patients who had undergone thermal ablation (RFA, MWA, or combined modalities) for primary PTC and subsequently required thyroidectomy (total *n* = 45). The clinical context of these studies is fundamentally different from Group A: ablation was performed as an alternative to surgery for primary malignancy, and surgery was driven primarily by oncological concerns (residual tumor, lymph node metastases, incomplete ablation, or tumor progression), rather than by benign nodule regrowth.

Intraoperatively, perithyroidal adhesions were consistently noted. Ma et al. [25] reported adhesion of postablation lesions to the strap muscles in 6/12 cases (50%), cauterization of the strap muscles in 5/12 (42%), and involvement of the RLN in 1/12 (8%). Sun et al. [26] found marked swelling of the strap muscles in 22/28 ablated nodules (78.6%) intraoperatively. Ding et al. [24] reported strap muscle adherence in 3/12 cases (25%), without significant impact on surgical difficulty. Postoperatively, Ma et al. [25] documented RLN palsy in 1/12 patients (8.3%), attributable to RLN involvement at ablation, and hoarseness was reported in 2/21 patients (9.5%) by Sun et al. [26]—both related to intraoperative RLN adherence or invasion at the ablation site. Ding et al. [24] reported no postoperative complications (Table 2).

**TABLE 2:** Surgical outcomes after thyroidectomy following prior thermal ablation, stratified by clinical scenario.

First author	Year	operative time	Adhesions/intraoperative findings	RLN palsy (transient) [per patient]	Hematoma [per patient]	Hypoparathyroidism [per patient]	Other surgical findings
*Group A—Benign/indeterminate nodules*							
Piana et al.	2012	NR	NR	0/22 pt (0%)	0/22 pt (0%)	0/22 pt (0%)	None
Russotto et al.	2024	NR	NR	0/7 pt (0%)	0/7 pt (0%)	0/7 pt (0%)	None
Dobrinja et al.	2015	98.5 ± 6.5 min (both cases)	No perithyroidal fibrosis	0/2 pt (0%)	0/2 pt (0%)	0/2 pt (0%)	None
Zhang et al.	2026	50 min median (total TT)	Fibrosis/scarring noted; pericapsular inflammation 48.4%	3/31 pt (9.7%)	2/31 pt (6.5%)	2/31 pt (6.5%)	5/47 NAR LOS (10.6%)
Hussein et al.	2024	110 min (vs 92 min controls, *p* = 0.40)	NR	2/23 pt (8.7%)	1/23 pt (4.3%)	0/23 pt (0%)	3/23 signal loss (13%)
Kuo et al.	2024	99.5 vs. 69.5 min (*p* = 0.01)	MAS ≥ 3: ant 50%, post 35%; TDS 9 vs. 6 (*p* < 0.001)	0/28 NAR (0%)	0/20 pt (0%)	0/8 bilateral pt (0%)	Incidental PTX 3/28 lobes (10.7%)
*Group B—Primary papillary thyroid carcinoma*							
Ding et al.	2022	NR	Strap muscle adherence 3/12 (25%)	0/12 pt (0%)	0/12 pt (0%)	0/12 pt (0%)	No major complication
Ma et al.	2018	NR	Strap muscle 6/12 (50%); strap cauterization 5/12; RLN involved 1/12	1/12 pt (8.3%)	NR	NR	RLN 1/12 (8.3%)
Sun et al.	2020	NR	Strap muscle swollen 22/28 nodules (78.6%)	2/21 pt (9.5%)	NR	NR	Hoarseness 2 pt (RLN adherence/invasion)

*Note:* Outcomes are reported per patient unless otherwise specified.

Abbreviations: ant = anterior; LNM = lymph node metastasis; LOS = intraoperative loss of signal; MAS = macroscopic adhesion score; NAR = nerves at risk; NR = not reported; post = posterior; pt = per patient; PTH = parathyroid hormone; PTX = parathyroidectomy; RLN = recurrent laryngeal nerve; TDS = thyroidectomy difficulty scale; TT = total thyroidectomy.

#### 3.4.2 Histopathological Findings—PTC Group

All three Group B studies reported histopathological findings. The most salient observation was the high rate of residual tumor and unaddressed lymph node metastases at surgical pathology, reflecting both the biology of PTC and the technical limitations of focal ablation as monotherapy for malignant thyroid disease.

Ma et al. [25] confirmed residual PTC in all 12 patients (100%) by histopathology, with lymph node metastases in 8/12 (66.7%) and multifocality in 9/12 (75%). Sun et al. [26] similarly found residual PTC in all 21 patients (100%), bilateral cancer in 7/21 (33.3%), central lymph node metastases in 10/21 (47.6%), and lateral metastases in 2/21 (9.5%). Additionally, FNA results were inconsistent with postoperative pathology in 5/32 nodules (15.6%), all of which were ≤ 1 cm, suggesting sampling limitations for small lesions. In contrast, Ding et al. [24]—who specifically selected unifocal, intrathyroidal, clinically node‐negative, low‐risk micro‐PTC—demonstrated complete local tumor elimination in all eight unifocal micro‐PTC cases, supporting the principle that meticulous patient selection is critical to ablation outcomes in the malignant setting.

These divergent findings between the three Group B studies are largely explained by differences in patient selection: Ma et al. [25] and Sun et al. [26] included patients with higher‐risk features (lesions > 1 cm, multifocal disease, or pre‐existing lymph node metastasis concerns), while Ding et al. [24] strictly applied low‐risk criteria. These data should not be interpreted as evidence that ablation is uniformly ineffective for PTC, but rather as a reminder that appropriate patient selection is the cornerstone of oncological adequacy in this setting (Table 3).

**TABLE 3:** Histopathological findings in postablation thyroid specimens, stratified by clinical scenario.

First author	Year	Chronic inflammation	Coagulative necrosis	Fibrosis/hyalinization	Residual tumor	Final diagnosis/IHC	Key observations
*Group A—Benign/indeterminate nodules*							
Piana et al.	2012	All (100%)	Variable (early: acute; late: chronic)	All (100%): dark fibrous scar	0/22 (0%)	None in LA‐treated nodules; 2 incidental micro‐PTC (distinct sites)	Fibrous capsule; carbonization; necrotic debris; temporal evolution well‐described
Russotto et al.	2024	5/7 (71%)	0/7 acute; chronic NR	5/7 (71%): sclerosis	0/7 (0%)	All IHC neg (Gal‐3, BRAF V600E, p53, HBME‐1); Ki‐67 < 3%	Hemorrhage 6/7; oncocytic cells 4/7; no capsular/vascular invasion
Dobrinja et al.	2015	NR	NR	Central hyaline sclerosis	0/2 (0%)	Case 1: follicular carcinoma (capsular inv. preserved); Case 2: follicular neoplasm UMP	RFA sclerosis did not alter capsule integrity; diagnostic assessment feasible
Zhang et al.	2026	19.2% mean (quantitative)	Chronic inflammation + hemorrhage 48.4%	19.2% mean sclerosis; 66.1% residual viability	NR	2 incidental micro‐PTC + 1 medullary CA (all outside ablated zone)	Sclerosis only factor associated with complications (*p* = 0.008); maturation index ↑ with time
Hussein et al.	2024	NR	NR	NR	NR	All confirmed benign on final pathology	No malignancy; coagulative necrosis → fibrosis confirmed pathologically
Kuo et al.	2024	16/20 (80%)	17/20 (85%): chronic; 1/20 acute	Acellular hyalinization 19/20 (95%)	3/20 (15%)	3 follicular carcinoma; 2 follicular UMP; 1 FND + incidental micro‐PTC	Atypical follicular cells 6/20 (30%); capsular invasion assessment challenging in 5/8 follicular neoplasms
*Group B—Primary papillary thyroid carcinoma*							
Ding et al.	2022	NR	Chronic inflammation (all disease‐free cases)	Fibrosis (all)	0/8 unifocal micro‐PTC; 2/4 PTC > 1 cm or multifocal	No residual CA in 8/8 unifocal micro‐PTC after MWA	Granuloma 1 case; Hashimoto thyroiditis 1 case
Ma et al.	2018	NR	NR	NR	12/12 (100%)	Residual PTC all cases; LNM 8/12 (66.7%); multifocal 9/12 (75%)	Coagulative necrosis around needle track; pTNM T1a–T3b
Sun et al.	2020	NR	NR	NR	21/21 (100%)	Bilateral CA 7/21 (33.3%); central LNM 10/21 (47.6%); lateral LNM 2/21 (9.5%)	Necrotic PTMC on RFA‐side; residual PTC contralateral; FNA incorrect in 5/32 nodules (15.6%)

Abbreviations: CA = carcinoma; FND = follicular nodular disease; Gal‐3 = galectin‐3; IHC = immunohistochemistry; LNM = lymph node metastasis; NR = not reported; UMP = uncertain malignant potential.

##### 3.4.2.1 Oncological Follow‐Up in Group B

None of the three Group B studies reported structured postoperative oncological follow‐up. Available data are essentially limited to surgical pathology; no study provided information on postthyroidectomy serum thyroglobulin levels, radioactive iodine administration (indications, dose, timing), imaging‐based surveillance, or rates and timing of structural or biochemical recurrence. In addition, the interval between ablation and salvage surgery was very short in two reports (median 30 days [26]; median 6.6 months [24]), making it impossible to evaluate the long‐term oncological effect of ablation prior to definitive surgery. This lack of systematic postoperative oncological follow‐up constitutes a major evidence gap: on the basis of current data, no robust conclusions can be drawn regarding the effectiveness of surgical salvage in achieving durable oncological control after thermal ablation for primary PTC.

## 4 Discussion

This systematic review provides a structured synthesis of surgical, strategic, and histopathological outcomes in patients undergoing thyroidectomy after prior thermal ablation of thyroid nodules. Across nine retrospective studies including 150 patients, postablation thyroidectomy appears technically feasible and, in experienced centers, is associated with low rates of permanent complications. At the same time, several consistent signals and important evidence gaps emerge, particularly regarding surgical risk, surgical strategy, and pathological or oncological assessment.

A distinctive feature of the thyroid setting, compared with ablation in the liver, lung, kidney, or uterus, is that delayed surgery almost always reflects true treatment failure rather than a planned step within an integrated ablation–resection pathway. In the present cohort, thyroidectomy was generally undertaken because ablation did not provide durable control—such as incomplete volume reduction, regrowth after an initial response, persistent or recurrent symptoms, discordant or upgraded cytology, or oncological inadequacy in malignant disease. This implies that our data describe a selected subset of failures emerging from a much broader ablated population and that the true burden of postablation surgery is likely underestimated, as not all centers systematically track or report such cases.

Across Group A (benign or indeterminate nodules), transient RLN palsy ranged from 0% to 9.7%, transient hypoparathyroidism from 0% to 6.5%, and cervical hematoma requiring reoperation from 0% to 6.5%; no permanent RLN palsy or permanent hypoparathyroidism was reported [11, 12, 20, 21, 22, 23]. On superficial reading, the numerous zero‐event series might suggest that prior ablation does not materially increase major complication rates compared with primary thyroidectomy. However, when pooled qualitatively, the broad ranges (0% to almost 10% for transient RLN dysfunction, 0% to approximately 6% to 7% for transient hypoparathyroidism and hematoma) are too wide to be considered reassuring and likely reflect small sample sizes, heterogeneous denominators, variable follow‐up, and publication bias toward high‐volume endocrine surgery centers.

### 4.1 Surgical Risk and Complexity

The most consistent signal across Group A is an increase in surgical complexity attributable to ablation‐induced fibrotic remodeling, while the impact on complication rates remains uncertain. The two methodologically stronger studies [11, 12] reported significantly higher TDS scores, more severe macroscopic adhesion scores, and longer operative times in post‐ablation thyroidectomies compared with contemporaneous non‐ablated controls [27, 28]. This is mechanistically plausible: thermal energy induces coagulative necrosis, chronic inflammation, and progressive fibrosis, tethering the thyroid capsule and nodule to the strap muscles, trachea, RLN, and parathyroid glands, thereby blurring anatomical planes.

Despite this clear increase in technical difficulty, the effect on surgical risk is not well quantified. Hussein et al. [23], in an explicitly high‐volume setting, found no statistically significant difference in overall complication rates between post‐RFA and control thyroidectomy (17.4% vs. 12.3%), whereas Zhang et al. [11] observed a numerically higher but non‐significant overall complication rate (22.6% vs. 12.9%) in postablation patients. All nine series are small, only two include matched controls, and none use adjusted analyses; thus, the true incremental risk of temporary or permanent complications attributable to ablation cannot be reliably estimated.

The absence of permanent RLN palsy and permanent hypoparathyroidism in Group A is reassuring but must be interpreted in context. Most series originate from specialized or high‐volume endocrine surgery centers, even when this status is not explicitly stated. In such environments, baseline rates of permanent RLN injury and permanent hypoparathyroidism after primary total thyroidectomy are typically in the range of 0.3%–1% and 0%–3%, respectively [13, 14, 15, 29]. Whether postablation thyroidectomy genuinely maintains these benchmarks, or whether small numbers have simply failed to capture rare events, remains unresolved.

Within this limited evidence base, the finding by Zhang et al. [11] that the degree of sclerosis within the ablated nodule correlated with postoperative complications suggests that qualitative and quantitative features of fibrosis, rather than prior ablation per se or elapsed time alone, may be key determinants of operative risk. However, the sclerosis score was exploratory, derived from a single cohort of 31 patients and not externally validated; at present, it should be viewed as hypothesis‐generating only.

### 4.2 Surgical Strategy

The data on how surgeons actually approach and plan postablation thyroidectomy are remarkably sparse and heterogeneous.

With regard to the extent of resection, most Group A studies did not systematically specify whether lobectomy or total thyroidectomy was performed. Kuo et al. [12] was the only study to report this clearly, indicating that 12 of 20 patients (60%) underwent hemithyroidectomy and 8 (40%) total thyroidectomy, as inferred from 28 lobes at risk. In the remaining Group A and all Group B studies, total thyroidectomy is either implied or not distinguished from lobectomy, preventing robust assessment of whether and how often surgeons purposely limit the extent of surgery after failed ablation. No study described the use of staged thyroidectomy (two‐stage completion procedures), leaving open whether this strategy was never used or simply under‐reported in particularly complex cases.

The adoption of IONM and NIRF was similarly incomplete. Only three Group A studies [11, 12, 23] explicitly or implicitly documented IONM [30], with intraoperative LOS in roughly 10%–13% of nerves at risk in the ablated groups; the other six studies, including all three PTC series, did not mention neuromonitoring. None of the nine studies reported the use of NIRF imaging for parathyroid identification or perfusion assessment, despite its increasing availability in high‐volume endocrine units and potential relevance in scarred operative fields. These gaps limit our ability to assess whether routine IONM and NIRF might mitigate risk in postablation thyroidectomy and to what extent current practice already incorporates these adjuncts.

Several other components of surgical strategy were poorly captured. Presurgical nodule VRR—a potential indicator of ablative efficacy that could influence both technical difficulty and decision‐making—was never reported as an aggregate quantitative variable, although “suboptimal VRR” was cited qualitatively as one indication for surgery in two studies. Pre‐operative ultrasound characteristics were frequently described as “suspicious,” but no series applied a formal TIRADS or equivalent classification at the time of surgical decision. The time interval between ablation and surgery, which might affect the maturity and density of fibrosis, was highly variable, ranging from days to more than 10 years, and reported using non‐uniform metrics. None of the studies specified the cervical access route (standard midline versus lateral “back‐door” approach to the strap muscles), and no series reported the use of minimally invasive, endoscopic, or robotic techniques; all operations appear to have been performed by conventional open surgery. The lack of detail on access and technique precludes any correlation between specific operative strategies and surgical difficulty or risk in the postablation setting.

### 4.3 Pathology and Oncologic Outcomes

From a pathological perspective, postablation thyroidectomy specimens in benign and indeterminate nodules show a characteristic but variably expressed pattern of chronic inflammation, coagulative necrosis, acellular hyalinization, and residual viable parenchyma. Three studies [11, 12, 20] provided detailed descriptions, including temporal evolution and quantitative estimates, whereas others limited reporting to final diagnoses or a few morphological descriptors. These data confirm that fibrosis and hyalinization are near‐universal consequences of ablation but also highlight substantial interpatient heterogeneity that is not yet predicted by simple clinical or imaging features.

Preoperative ultrasound and presurgical VRR, which might theoretically anticipate the degree of histological remodeling, were not systematically coded or correlated with pathology. As a result, it remains unknown whether specific baseline ultrasound patterns, nodule composition, or degrees of volume reduction predict distinct histological substrates (e.g., highly hyalinized versus predominantly viable nodules) and whether such substrates, in turn, influence operative complexity or complication risk.

Treatment‐related cytological and histological atypia, particularly in follicular‐patterned neoplasms, raise significant diagnostic challenges. Nuclear enlargement, chromatin clearing, and clear‐cell change may mimic papillary‐type nuclear features, while stromal hyalinization and fragmentation may either obscure or simulate capsular and vascular invasion. Immunohistochemical and molecular adjuncts (e.g., NRAS Q61R, BRAF V600E, galectin‐3, HBME‐1) have proven useful in resolving equivocal cases [12, 21]. No study documented carcinoma arising within the ablated zone [31, 32], and incidental microcarcinomas identified at sites distant from the treated nodule were consistent with background prevalence; however, small sample sizes and limited follow‐up do not allow definitive exclusion of rare events.

In the malignant setting (Group B), where ablation was used as the primary treatment for PTC, surgical specimens consistently showed high rates of residual tumor and lymph node metastases, especially in series with less restrictive selection criteria [25, 26]. In contrast, a small cohort of strictly selected unifocal, intrathyroidal micro‐PTC treated by ablation showed complete local tumor elimination at the time of surgery [24]. These divergent findings strongly support the notion that oncological adequacy is primarily determined by patient selection, rather than by the ablation technique alone [33].

Crucially, postthyroidectomy oncological follow‐up in Group B was almost entirely absent. None of the three PTC series provided systematic data on postoperative thyroglobulin levels, radioactive iodine therapy, imaging surveillance, or structural or biochemical recurrence. Moreover, the intervals between ablation and surgery were often short (weeks to a few months), precluding any assessment of the long‐term effect of ablation alone on disease control. Consequently, while the available data confirm that many patients referred for surgery after ablation harbor substantial residual PTC and unaddressed nodal disease, the long‐term oncological trajectory after salvage thyroidectomy remains undefined, and no conclusions can be drawn regarding whether prior ablation compromises, improves, or leaves unchanged prognosis relative to standard upfront surgery.

These observations are consistent with current guidelines, which continue to recommend surgery as the standard initial treatment for operable PTC and restrict thermal ablation to carefully selected low‐risk micro‐PTC in patients who decline or are unfit for surgery [33, 34, 35]. The present review does not alter these recommendations but adds important contextual information on the surgical and pathological substrate encountered when ablation is used outside ideal selection criteria and definitive surgery is subsequently required.

### 4.4 Temporal Trends, Center Volume, and Generalisability

The publication timeline of the nine studies shows a clear temporal trend, with only two reports between 2012 and 2021 and five between 2022 and 2026, including three published in 2024 alone. This pattern reflects the progressive incorporation of thyroid ablation into routine practice and the delayed emergence of sufficiently large surgical rescue cohorts to warrant dedicated analysis.

Most series were conducted in specialized or high‐volume endocrine surgery centers, although this status was explicitly declared in only one study and inferred in another. Several Italian and Chinese institutions are recognized referral centers for thyroid surgery, yet annual volumes were generally not quantified. Concentration of cases in expert centers likely contributes to the very low rate of permanent complications and raises questions regarding generalisability: outcomes observed in high‐volume, IONM‐equipped, pathologically specialized units may not be reproducible in lower‐volume settings with fewer resources or less specific expertise.

### 4.5 Methodological Considerations and Nature of the Evidence

A central methodological point is that the evidence on thyroidectomy after thermal ablation is intrinsically limited: all included studies are retrospective, sample sizes are small, denominators and outcome definitions vary, and only two series include matched non‐ablated controls. In addition, selection bias (only failures proceeding to surgery are captured) and likely publication bias toward high‐volume endocrine centers further restrict generalizability. Under these circumstances, our review should be interpreted primarily as an evidence‐mapping exercise rather than a definitive answer to a focused clinical question.

In line with the logic of scoping reviews and other Joanna Briggs Institute–endorsed evidence synthesis methodologies, the present work systematically describes how, where, and under which conditions thyroidectomy after ablation has been reported, which surgical and pathological issues recur across series, and which dimensions remain poorly documented. This perspective explains the intentionally descriptive tone of our results and discussion and justifies our decision not to perform meta‐analysis and not to overinterpret the wide ranges and multiple zero‐event reports for key complications such as RLN injury, hypoparathyroidism, and cervical hematoma.

Accordingly, we deliberately avoid strong, prescriptive clinical statements. Our conclusions are framed around three main messages supported by the available data: (i) postablation thyroidectomy is feasible and appears to carry low permanent morbidity in experienced endocrine centers; (ii) prior ablation consistently increases operative complexity and may challenge parathyroid preservation and pathological assessment of follicular‐patterned neoplasms; and (iii) current evidence is insufficient to define the true incremental surgical risk, the optimal operative strategy, or the long‐term oncologic impact of ablation in PTC. From a methodological standpoint, these observations underscore the need for prospective, multicenter, ideally comparative, studies explicitly designed to address surgical risk, strategy, and oncologic outcomes in this setting.

### 4.6 Limitations

The limitations of this review are substantial. All included studies were retrospective, with small sample sizes and heterogeneous designs, resulting in limited statistical power and susceptibility to random error and reporting bias. Only two studies included matched nonablated controls, and none used adjusted analyses. Ablation modalities, indications, ablation‐to‐surgery intervals, definitions of “surgical difficulty,” and outcome metrics were highly variable, precluding formal meta‐analysis.

Reporting of key variables was incomplete and inconsistent. Denominators (per patient, per lobe, per nerve at risk) varied; follow‐up for late complications was often short or unspecified; and critical elements of surgical strategy—extent and staging of resection, IONM, and NIRF protocols, preoperative VRR, structured US classification, access route—were frequently absent. Selection bias is intrinsic: patients requiring surgery after ablation are not representative of all ablated patients, and publication bias probably favors series from high‐volume centers with specific interest in complex thyroid surgery. In the PTC group, the complete lack of systematic post‐thyroidectomy oncological follow‐up is a major limitation that prevents any meaningful assessment of long‐term cancer control after salvage surgery.

Taken together, these constraints preclude definitive conclusions and highlight the urgent need for prospective, multicenter, comparative studies with standardized ablation and surgical protocols, matched control cohorts, robust endpoint definitions, and sufficiently long follow‐up, explicitly addressing surgical risk, surgical strategy, and pathological and oncological outcomes.

## 5 Conclusion

This systematic review shows that thyroidectomy after prior thermal ablation is technically feasible and, in high‐volume endocrine centers, associated with low rates of permanent morbidity. The reported series did not document permanent RLN palsy or permanent hypoparathyroidism, but wide ranges for transient complications and frequent zero‐event reports reflect small, heterogeneous cohorts and do not define robust safety benchmarks. Regarding surgical strategy, prior ablation is consistently linked to greater operative complexity—more adhesions, higher difficulty scores, and longer operative times—whereas data on extent of resection, staged procedures, use of neuromonitoring and fluorescence, and access routes are sparse, preventing firm recommendations on optimal operative planning. Histopathologically, fibrosis, hyalinization, and treatment‐related atypia complicate the evaluation of follicular‐patterned neoplasms and mandate explicit communication of ablation history and judicious use of ancillary markers; current data do not support ablation‐induced malignant transformation, although rare events cannot be excluded. In patients ablated for primary PTC, small series—often with suboptimal selection—show high rates of residual tumor and nodal disease at thyroidectomy, and virtually no structured post‐thyroidectomy oncological follow‐up, so the long‐term efficacy of surgical salvage remains undefined and current guidelines favoring surgery as first‐line treatment for operable PTC are unchanged. Overall, thyroidectomy after thermal ablation appears safe in expert hands but is more complex than primary surgery, and the existing, center‐biased evidence is insufficient to set definitive standards for surgical risk, strategy, or oncological outcomes; well‐designed prospective multicenter studies are needed.

## Author Contributions

F.B. and G.D. conceived the study. F.B., F.P., and B.S. designed the review protocol, performed the literature search, screened studies, extracted data, and drafted the manuscript. G.D., C.C., and L.F. supervised the project and critically revised the manuscript for important intellectual content. All authors contributed to data interpretation, reviewed the final manuscript, and approved the submitted version.

## Funding

The authors have nothing to report.

## Ethics Statement

The authors have nothing to report.

## Consent

The authors have nothing to report.

## Conflicts of Interest

Gianlorenzo Dionigi reports a consultancy contract with Beckdal (Shanghai) Med Tech Co. Ltd., Wenxiang East Road, Songjiang District, Shanghai, China. The other authors declare no conflicts of interest.

## Supporting information


**Appendix S1:** Full electronic search strategy.
**Appendix S2:** Newcastle–Ottawa Scale: numerical scores.
**Table S2a:** Newcastle–Ottawa Scale numerical scores for all included studies.
**Table S2b:** Item‐level justifications for NOS scores.

## Data Availability

The data that support the findings of this study are available from the corresponding author upon reasonable request.
