# Supplementary material for: Surgery After Thermal Ablation of Thyroid Nodules: A Systematic Review of Surgical Risk, Strategy and Pathological Findings
Source: Head Neck. 2026 Sep 27;48(12):3480–94. doi: 10.1002/hed.70487 (PMC13755740; doi:10.1002/hed.70487)
Supplement: Supplementary file 1 [file HED-48-3480-s001.docx]

**SUPPLEMENTARY MATERIAL**

**Surgical and Pathological Outcomes of Thyroidectomy Following Prior Thermal Ablation**

*A Systematic Review with Descriptive Synthesis*

**Contents of this Supplementary File:**

• Appendix S1 — Full Electronic Search Strategy (PubMed, EMBASE, Cochrane)

• Appendix S2 — Newcastle-Ottawa Scale: Numerical Scores with Item-Level Justifications

# Appendix S1 — Full Electronic Search Strategy

**PubMed / MEDLINE**

1. "thyroid nodule"[MeSH Terms] OR "thyroid nodule"[tiab] OR "thyroid nodules"[tiab]

2. "thyroid neoplasms"[MeSH Terms] OR "papillary thyroid carcinoma"[tiab] OR "thyroid cancer"[tiab]

3. #1 OR #2

4. "radiofrequency ablation"[MeSH Terms] OR "radiofrequency ablation"[tiab] OR "RFA"[tiab]

5. "laser ablation"[tiab] OR "laser therapy"[MeSH Terms] OR "interstitial laser"[tiab]

6. "microwave ablation"[tiab] OR "thermal ablation"[tiab] OR "percutaneous ablation"[tiab]

7. #4 OR #5 OR #6

8. "thyroidectomy"[MeSH Terms] OR "thyroid surgery"[tiab] OR "thyroid resection"[tiab]

9. "surgical outcome"[tiab] OR "postoperative complication"[tiab] OR "histopathology"[tiab] OR "pathology"[tiab]

10. #3 AND #7 AND #8

11. #10 AND (#9)

Filters: Humans; English; [DATE FILTER — to be applied]

**EMBASE**

1. 'thyroid nodule'/exp OR thyroid nodule:ti,ab

2. 'thyroid carcinoma'/exp OR 'papillary thyroid carcinoma':ti,ab OR 'thyroid cancer':ti,ab

3. #1 OR #2

4. 'radiofrequency ablation'/exp OR 'radiofrequency ablation':ti,ab OR 'RFA':ti,ab

5. 'laser ablation':ti,ab OR 'laser therapy'/exp OR 'interstitial laser':ti,ab

6. 'microwave ablation':ti,ab OR 'thermal ablation':ti,ab OR 'percutaneous ablation':ti,ab

7. #4 OR #5 OR #6

8. 'thyroidectomy'/exp OR 'thyroid surgery':ti,ab

9. 'surgical outcome':ti,ab OR 'postoperative complication':ti,ab OR 'histopathology':ti,ab

10. #3 AND #7 AND #8

11. #10 AND #9

Filters: Human; English; [DATE FILTER — to be applied]

**Cochrane Library (CENTRAL)**

MeSH descriptor: [Thyroid Nodule] explode all trees

MeSH descriptor: [Thyroid Neoplasms] explode all trees

"radiofrequency ablation" OR "laser ablation" OR "microwave ablation" OR "thermal ablation"

"thyroidectomy" OR "thyroid surgery"

Combine #1 OR #2 AND #3 AND #4

[DATE FILTER — to be applied]

# Appendix S2 — Newcastle-Ottawa Scale: Numerical Scores

## S2.1 NOS Cohort Scale — Scoring Criteria

The Newcastle-Ottawa Scale (NOS) for cohort studies assigns up to 9 'stars' (★) distributed across three domains: Selection (max 4★), Comparability (max 2★), and Outcome (max 3★). Quality thresholds: ≥7★ = Low risk of bias; 5–6★ = Moderate; ≤4★ = High. The individual items scored are as follows:

**SELECTION (max 4★)**

S1 — Representativeness of the exposed cohort: ★ if the cohort is representative of the ablated thyroid population (e.g., consecutive patients); ☆ if selected/referred cases only.

S2 — Selection of the non-exposed cohort: ★ if a non-ablated comparator group is drawn from the same institution or community; ☆ if no comparator group is present (scored 0 by definition for single-arm series).

S3 — Ascertainment of exposure (ablation): ★ if confirmed by secured records (institutional registry, operative notes); ☆ if by self-report or assumption.

S4 — Demonstration that outcome was not present at start of study: ★ if malignant diagnosis before ablation was an explicit exclusion criterion and histopathological verification available; ☆ if not verifiable.

**COMPARABILITY (max 2★)**

C1 — Comparability on the basis of the most important confounding factor (prior ablation vs. no ablation): ★ if analysis controls for or matches on the primary confounding factor; ☆ if no comparator or no adjustment.

C2 — Comparability on any additional factor (age, nodule size, ablation-to-surgery interval): ★ if additional factor controlled for; ☆ otherwise.

Note: for single-arm series without any comparator group, both C1 and C2 are scored 0 (maximum achievable = 7/9).

**OUTCOME (max 3★)**

O1 — Assessment of outcome: ★ if assessed by independent/blinded assessment (e.g., dedicated pathologist, independent surgeon performing laryngoscopy, or standardised protocol); ☆ if by self-report or non-standardised assessment.

O2 — Follow-up long enough for outcomes to occur: ★ if ablation-to-surgery interval is reported and adequate for the outcome assessed (≥3 months for complications; ≥12 months for long-term histopathological remodelling); ☆ if not reported or inadequate.

O3 — Adequacy of follow-up of cohorts: ★ if complete follow-up or loss-to-follow-up <20% with justification; ☆ if follow-up not described or loss-to-follow-up >20%.

## S2.2 NOS Summary Scores

Table S2a presents the NOS numerical scores for each included study. Studies are grouped by clinical scenario (Group A: benign/indeterminate nodules; Group B: primary PTC). Individual item scores (0 or 1 star per item) are shown alongside domain subtotals and overall total scores. Quality category thresholds: Low RoB (green) = ≥7/9; Moderate RoB (yellow) = 5–6/9; High RoB (red) = ≤4/9.

**Table S2a. Newcastle-Ottawa Scale numerical scores for all included studies.**

| **Study** | **Year** | **SELECTION (max ★★★★)** | | | | | **COMPARABILITY (max ★★)** | | | **OUTCOME (max ★★★)** | | | | **TOTAL /9** | **Quality** |
| --- | --- | --- | --- | --- | --- | --- | --- | --- | --- | --- | --- | --- | --- | --- | --- |
|  |  | **S1 Represent.** | **S2 Control sel.** | **S3 Exposure asc.** | **S4 Outcome-free at start** | **Selection subtotal** | **C1 Primary factor** | **C2 Addit. factor** | **Comp. subtotal** | **O1 Outcome asc.** | **O2 Follow-up length** | **O3 Follow-up adequacy** | **Outcome subtotal** |  |  |
| **Group A — Benign / Indeterminate Nodules** | | | | | | | | | | | | | | | |
| **Piana et al.** | 2012 | **★** 1/1 | ☆ 0/1 | **★** 1/1 | **★** 1/1 | **★★★**☆ 3/4 | ☆ 0/1 | ☆ 0/1 | ☆☆ 0/2 | **★** 1/1 | **★** 1/1 | ☆ 0/1 | **★★**☆ 2/3 | **5/9** | **Moderate RoB** |
| **Russotto et al.** | 2024 | **★** 1/1 | ☆ 0/1 | **★** 1/1 | **★** 1/1 | **★★★**☆ 3/4 | ☆ 0/1 | ☆ 0/1 | ☆☆ 0/2 | **★** 1/1 | ☆ 0/1 | ☆ 0/1 | **★**☆☆ 1/3 | **4/9** | **High RoB** |
| **Dobrinja et al.** | 2015 | ☆ 0/1 | ☆ 0/1 | **★** 1/1 | **★** 1/1 | **★★**☆☆ 2/4 | ☆ 0/1 | ☆ 0/1 | ☆☆ 0/2 | **★** 1/1 | **★** 1/1 | ☆ 0/1 | **★★**☆ 2/3 | **4/9** | **High RoB** |
| **Zhang et al.** | 2026 | **★** 1/1 | **★** 1/1 | **★** 1/1 | **★** 1/1 | **★★★★** 4/4 | **★** 1/1 | ☆ 0/1 | **★**☆ 1/2 | **★** 1/1 | **★** 1/1 | **★** 1/1 | **★★★** 3/3 | **8/9** | **Low RoB** |
| **Hussein et al.** | 2024 | **★** 1/1 | **★** 1/1 | **★** 1/1 | **★** 1/1 | **★★★★** 4/4 | **★** 1/1 | ☆ 0/1 | **★**☆ 1/2 | **★** 1/1 | **★** 1/1 | **★** 1/1 | **★★★** 3/3 | **8/9** | **Low RoB** |
| **Kuo et al.** | 2024 | **★** 1/1 | **★** 1/1 | **★** 1/1 | **★** 1/1 | **★★★★** 4/4 | **★** 1/1 | ☆ 0/1 | **★**☆ 1/2 | **★** 1/1 | **★** 1/1 | **★** 1/1 | **★★★** 3/3 | **8/9** | **Low RoB** |
| **Group B — Primary Papillary Thyroid Carcinoma** | | | | | | | | | | | | | | | |
| **Ding et al.** | 2022 | **★** 1/1 | ☆ 0/1 | **★** 1/1 | ☆ 0/1 | **★★**☆☆ 2/4 | ☆ 0/1 | ☆ 0/1 | ☆☆ 0/2 | **★** 1/1 | **★** 1/1 | **★** 1/1 | **★★★** 3/3 | **5/9** | **Moderate RoB** |
| **Ma et al.** | 2018 | ☆ 0/1 | ☆ 0/1 | **★** 1/1 | ☆ 0/1 | **★**☆☆☆ 1/4 | ☆ 0/1 | ☆ 0/1 | ☆☆ 0/2 | **★** 1/1 | ☆ 0/1 | ☆ 0/1 | **★**☆☆ 1/3 | **2/9** | **High RoB** |
| **Sun et al.** | 2020 | ☆ 0/1 | ☆ 0/1 | **★** 1/1 | ☆ 0/1 | **★**☆☆☆ 1/4 | ☆ 0/1 | ☆ 0/1 | ☆☆ 0/2 | **★** 1/1 | ☆ 0/1 | ☆ 0/1 | **★**☆☆ 1/3 | **2/9** | **High RoB** |

*Abbreviations: S1–S4 = Selection domain items; C1–C2 = Comparability domain items; O1–O3 = Outcome domain items. ★ = criterion met (1 point); ☆ = criterion not met (0 points). Colour coding for overall quality: green = Low RoB (≥7/9); yellow = Moderate RoB (5–6/9); red = High RoB (≤4/9). N/A: for single-arm series without a comparator group, S2 and C1/C2 are scored 0 by definition.*

## S2.3 Item-Level Justifications

Table S2b provides explicit justification for each NOS score assigned per study and per item. Scores were assigned independently by two reviewers; disagreements were resolved by consensus.

**Table S2b. Item-level justifications for NOS scores.**

| **Study** | **Year** | **NOS Item** | **Justification for score assigned** |
| --- | --- | --- | --- |
| **Piana et al.** | 2012 | **S1** | : Consecutive ablation patients from single centre; representative of treated population. |
|  |  | **S2** | : No non-ablated comparator group. |
|  |  | **S3** | : Exposure (LA) confirmed by institutional records. |
|  |  | **S4** | : Surgical specimens analysed; outcome not present at ablation. |
|  |  | **C1** | C2☆: No comparator group — comparability not assessable. |
|  |  | **O1** | : Histopathological assessment by dedicated pathologist. |
|  |  | **O2** | : Ablation-to-surgery interval documented (mean 38.6 mo); adequate for fibrosis assessment. |
|  |  | **O3** | : No standardised follow-up protocol post-surgery; loss-to-follow-up not reported. |
| **Russotto et al.** | 2024 | **S1** | : Consecutive patients from pathology unit over 5-year period. |
|  |  | **S2** | : No comparator group. |
|  |  | **S3** | : Ablation (LA) confirmed by institutional records. |
|  |  | **S4** | : All nodules pre-confirmed benign before ablation. |
|  |  | **C1** | C2☆: No comparator group. |
|  |  | **O1** | : IHC panel (Gal-3, HBME-1, BRAF V600E, p53, Ki-67) applied systematically. |
|  |  | **O2** | : Ablation-to-surgery interval not reported; follow-up duration unclear. |
|  |  | **O3** | : Retrospective; incomplete follow-up documentation. |
| **Dobrinja et al.** | 2015 | **S1** | : Only 2/6 Thy3 nodules required surgery — highly selected subgroup, not representative. |
|  |  | **S2** | : No comparator group. |
|  |  | **S3** | : RFA confirmed by institutional records. |
|  |  | **S4** | : Thy3 nodules confirmed without malignancy before ablation. |
|  |  | **C1** | C2☆: No comparator group. |
|  |  | **O1** | : Histopathology by 4 dedicated pathologists; IHC (HBME-1) and molecular analysis performed. |
|  |  | **O2** | : Mean interval 15 mo documented; follow-up 2 years. |
|  |  | **O3** | : Very small n=2; completeness of follow-up cannot be adequately assessed. |
| **Zhang et al.** | 2026 | **S1** | : All consecutive post-ablation thyroidectomies at single high-volume centre 2021–2025. |
|  |  | **S2** | : Contemporaneous non-ablated thyroidectomy cohort included (n=819). |
|  |  | **S3** | : Ablation modality (RFA/EtOH) confirmed by institutional registry. |
|  |  | **S4** | : Malignant diagnosis before ablation was an exclusion criterion. |
|  |  | **C1** | : Comparison performed against contemporaneous non-ablated cohort. |
|  |  | **C2** | : No matching or risk-adjustment performed; groups differ in baseline characteristics. |
|  |  | **O1** | : Blinded dual-pathologist semiquantitative morphometric assessment. |
|  |  | **O2** | : Mean ablation-to-surgery interval 43.9 mo; adequate for fibrotic remodelling. |
|  |  | **O3** | : Prospectively maintained registry; complete data for all 31 patients. |
| **Hussein et al.** | 2024 | **S1** | : All post-RFA thyroidectomies at single centre 2020–2022 (23/522 ablated = 4.4%). |
|  |  | **S2** | : Non-RFA thyroidectomy control group included (n=73). |
|  |  | **S3** | : RFA confirmed by institutional records. |
|  |  | **S4** | : All nodules benign on pre-RFA FNAB. |
|  |  | **C1** | : Univariate analysis showed groups comparable on most characteristics. |
|  |  | **C2** | : Age differed significantly (62.5 vs. 50.5 yrs, p<0.001); no propensity matching. |
|  |  | **O1** | : Postoperative laryngoscopy performed on all patients; pathology by dedicated team. |
|  |  | **O2** | : Median interval 6.7 mo; post-surgical follow-up documented. |
|  |  | **O3** | : Complete outcome data reported; loss-to-follow-up not documented. |
| **Kuo et al.** | 2024 | **S1** | : All patients from prospectively maintained institutional registry (20/445 = 4.5%). |
|  |  | **S2** | : Contemporaneous non-ablated benign nodule thyroidectomy control group (n=145). |
|  |  | **S3** | : RFA confirmed by institutional records; pre-RFA FNAB documented. |
|  |  | **S4** | : All confirmed benign before ablation; malignancy at surgery classified separately. |
|  |  | **C1** | : Validated objective scoring (TDS, MAS) used for intraoperative difficulty comparison. |
|  |  | **C2** | : No propensity matching; groups comparable on most baseline characteristics but selection criteria differ. |
|  |  | **O1** | : Intraoperative difficulty scored by ≥3 independent experienced surgeons; blinded dual-pathologist histopathology. |
|  |  | **O2** | : Mean interval 29.5 mo (range 3.9–129 mo); adequate follow-up. |
|  |  | **O3** | : Prospective registry; complete outcome data for all 20 patients. |
| **Ding et al.** | 2022 | **S1** | : Multicenter retrospective; 12 patients from 6 hospitals — broadly representative of PTC ablation failures. |
|  |  | **S2** | : No non-ablated PTC comparator group. |
|  |  | **S3** | : MWA confirmed; pre-ablation FNAB documented for all. |
|  |  | **S4** | : Primary outcome (residual PTC) is the reason for surgery — built-in ascertainment bias. |
|  |  | **C1** | C2☆: No comparator group. |
|  |  | **O1** | : Full histopathological evaluation at thyroidectomy; pTNM staging reported. |
|  |  | **O2** | : Median ablation-to-surgery interval 6.6 mo (range 0.4–21.9). |
|  |  | **O3** | : Complete pathological data for all 12 patients; no loss to follow-up. |
| **Ma et al.** | 2018 | **S1** | : Selected patients with suspicious post-ablation findings or LNM — highly selected, not representative. |
|  |  | **S2** | : No comparator group. |
|  |  | **S3** | : Ablation method documented (RFA/MWA/LA); FNAB pre-ablation in all. |
|  |  | **S4** | : Patients selected for surgery based on malignant post-ablation findings — outcome present at entry. |
|  |  | **C1** | C2☆: No comparator group. |
|  |  | **O1** | : Histopathology confirmed by dedicated pathologist; pTNM staging complete. |
|  |  | **O2** | : Ablation-to-surgery interval not systematically reported. |
|  |  | **O3** | : Retrospective chart review; completeness of follow-up not assessed. |
| **Sun et al.** | 2020 | **S1** | : Patients selected for surgery based on suspicious US/CT or LNM — not representative of all PTC ablation. |
|  |  | **S2** | : No comparator group. |
|  |  | **S3** | : RFA confirmed; pre-ablation FNAB documented. |
|  |  | **S4** | : Surgery performed because of suspected residual/recurrent disease — selection on outcome. |
|  |  | **C1** | C2☆: No comparator group. |
|  |  | **O1** | : Histopathology with pTNM staging; postoperative imaging documented. |
|  |  | **O2** | : Median interval only 30 days (range 6–368 d) — highly variable; no standardised follow-up. |
|  |  | **O3** | : Retrospective; completeness of follow-up unclear. |
